# Exploring the effect of the ‘Growing Together’ parenting education kit on early parenting - study protocol for a cluster randomised controlled trial

**DOI:** 10.1186/s40359-019-0314-z

**Published:** 2019-06-24

**Authors:** Touran Shafiei, Helen L. McLachlan, Jan M. Nicholson, Sarah Hay, Michelle Newton, Heather Grimes, Fiona McLardie-Hore, Della A. Forster

**Affiliations:** 1 Judith Lumley Centre, La Trobe University, George Singer Building, Kingsbury Dr, Bundoora, Victoria 3086 Australia; 2 School of Nursing and Midwifery, La Trobe University, George Singer Building, Kingsbury Dr, Bundoora, Victoria 3086 Australia; 3 Royal Women’s Hospital, Locked Bag 300 Grattan St & Flemington Rd, Parkville, Victoria 3052 Australia; 4 La Trobe Rural Health School, La Trobe University, Edwards Rd, Flora Hill, 3055 Australia

**Keywords:** Early parenting, Cluster randomised controlled trial, Motherhood, Attachment, Education

## Abstract

**Background:**

Significant gaps exist in education for prospective and new parents, especially for some of the most vulnerable families. Prospective parents would like more information during pregnancy to prepare them for parenting, and need access to trusted and quality information.

The Royal Women’s Hospital (the Women’s) in Melbourne, Australia, a large tertiary referral maternity hospital, developed a parenting education kit known as ‘*Growing Together*’. The kit, designed to guide prospective and new parents from conception until 1 year after birth, includes three components: an A4 sized book, a specifically designed ‘App’ and a children’s story book. We aim to evaluate the impact of the kit on a range of outcomes.

**Methods:**

A two-arm cluster randomised controlled trial will be used. Antenatal clinic days will be randomised to either the intervention or standard care arms. Women in the intervention arm receive the kit at their antenatal booking visit. Women in the standard care arm receive the standard information resources at the Women’s. Analyses will be by intention to treat. Inclusion criteria: primiparous women with adequate English-speaking ability and ≤ 30 weeks’ gestation at first pregnancy booking appointment.

The primary outcome of the study is the ‘experience of motherhood questionnaire’ (EMQ), a 20 item validated self-report measure, ranging from 0 to 80, with lower scores indicating better maternal health and wellbeing. To detect a 10% difference in new mothers scoring ≤40 between women who have received the kit (60%) and those who have not (50%), would require 408 per group (total of 816 women) with 95% confidence and 80% power. Allowing for loss to follow up, we aim to recruit 1000 mothers. Secondary outcomes include parents’ views and experiences of their care and of the kit during pregnancy and after the birth, parental attachment, knowledge, confidence, wellbeing and health-seeking behaviour; and emotional, developmental and physical health of the infant. Survey data will be collected from mothers at 2, 6 and 12 months postpartum and partners at 6 months.

**Discussion:**

This study will provide much needed high-level evidence on the impact of a comprehensive education resource for new parents.

**Trial registration:**

ANZCTRN12615000270516 - Retrospectively registered (23/03/2015); trial started on 16 March 2015.

## Background

There is growing recognition by health care providers that prospective parents would like more information during pregnancy to prepare them for parenting [1–3]. Once families leave hospital, the home environment is crucial to a child’s physical, cognitive and emotional development [4]. New mothers and their partners must learn ‘on the job’, often with little preparation beyond what they have observed of families around them and what they can gather from (sometimes conflicting) multiple information sources. Parents need direct access to quality information to help prepare them for their unique role in shaping their child’s future.

### Information needs of new parents

Information currently available for expectant and new parents varies widely in quality and accessibility, and may be sought from midwives, obstetricians, general practitioners (GPs), family, friends, Maternal and Child Health nurses (MCHNs), books, websites and social media. A national study on the information needs of Australian parents found that awareness of the range of available information was generally low, and that parents’ information needs were only partly addressed by the information they found [5]. Another Australian study involving antenatal educators and new mothers found that mothers rarely receive information beyond labour and birth management and breastfeeding in antenatal classes [6]. A recent systematic review and meta-synthesis of 12 qualitative studies reported that new parents often felt inadequately prepared for the early parenting period, and recommended that antenatal education classes place equal emphasis on the antenatal and postnatal periods [1]. A national review of the evidence for parenting interventions in Australia indicated that there are very few evidence-based programs which target child development, no programs for parents with disabilities or mental health issues, and no programs for teenage parents [7].

### Practical issues related to feeding and sleep

New parents also face many practical issues. Many experience anxiety and concern about infant feeding [8], the amount and pattern of their infant’s sleep [9], and about strategies for settling their infant and managing crying. Parents with concerns about their infant’s sleeping, feeding and crying are more likely to seek medical assistance in the belief that something is wrong [10], and are at increased risk of postnatal depression and fatigue [9, 11]. There is now growing evidence that anticipatory guidance provided in the early stages of parenthood helps parents to establish good infant sleep and feeding habits, reduces parent distress about normal infant crying, and reduces postnatal depression symptoms [12–14]. Despite this promising evidence, methods for ensuring the timely provision of such advice at a population level are lacking.

### The California kit for new parents

The California kit for New Parents was developed and piloted in the United States in 2001, and has since been distributed, at no charge, to more than two million Californian families [15]. The kit is made up of a DVD about the emotional and cognitive development of infants and children, a children’s book, and a parenting guide. It is available in six languages [15], and has been adopted by authorities in Arizona, Pennsylvania, Alabama and Kentucky [4].

Evaluations of the kit found that providing information (e.g. about antenatal care, early childhood learning and development, infant nutrition and access to services) to expectant and new parents resulted in significant improvements in parenting confidence, knowledge and behaviour [16]. Short- [4] and long-term [15] evaluations found that the kit had a positive impact on parents’ knowledge, attitudes and behaviours: 87% of mothers used the kit within 2 months of receiving it and 53% reported sharing it with their partner. A year after receiving the kit the majority of mothers reported using the kit multiple times; and 94% of mothers reported that the kit was helpful with a wide range of parenting issues. Mothers who received a kit demonstrated improved parenting practices in child development, infant feeding, early literacy, child safety, health care and access to local resources compared with mothers who did not receive a kit [4]. Knowledge gains were high for all parents who received a kit; with the highest among women who received the kit before the baby was born, and for women who spoke Spanish [4]. Parents reported liking the option of choosing from a variety of formats, identifying with people and parenting practices in the DVDs, and feeling empowered by seeing new ways of parenting.

### The ‘parenting kit – Growing Together – at the Women’s hospital

Based on the California kit for New Parents, a parenting kit was developed by the Royal Women’s Hospital (The Women’s) in Melbourne, Australia, a large tertiary referral maternity hospital, to guide prospective and new parents from conception until their child is 1 year of age, titled “*Growing Together*”. Drawing on existing research and the resources already available, *Growing Together* aims to inform parents about the ages and stages of development from conception to 12 months. It provides practical information—from a single authoritative, trusted and non-commercial source—about health care, health promotion, child care and social support and links to Victorian and Australian Government funded parenting services, resources and information.

The Women’s undertook a community engagement process to develop and pilot *Growing Together*, with the aim of then evaluating its impact to determine if a single kit, such as that being developed could meet the needs of all parents.

The content and presentation of the kit was determined in consultation with parents and families; participatory design was integral to its development. Community consultations with a diverse range of families shaped the design and content of the kit, so that messages about the importance of safe and secure relationships, encouraging learning and responding to the individual infant’s personality right from birth are communicated in accessible and empowering ways [17]. There was also a greater focus on parents’ mental health than traditional resources aimed at prospective and new parents. The kit design is generic and not focused only on the Women’s Hospital. Therefore, if it is shown to be acceptable and effective, it could be rolled out more broadly.

There are three parts to the *Growing Together* kit:

1. An A4 size book that covers the journey from conception to 1 year of age.
2. A phone ‘App’ comprising four modules – one each for preconception, pregnancy, parenting, and one for health professionals. The ‘App’ is interactive, and has push notifications, videos and links to various appropriate sites and many other features.
3. A children’s book written by Australian author Mem Fox, titled *Ten Little Fingers and Ten Little Toes,* is aimed at encouraging parents to read to their children, as well as containing what are considered to be important messages for parents.

The Growing Together book includes information about pregnancy and birth care options, different stages of the pregnancy and labour, care during pregnancy (including changing in the body and discomfort; relevant tests and screening; medicines, drugs and substances; important nutrients during pregnancy; when to contact hospital; emotional and mental health), labour and birth (e.g. preparing for labour and birth, when to go to hospital, pain relief in labour) and care after birth (e.g. breastfeeding; safe sleeping; sex and contraception; postnatal mental health).

Funding from the Victorian Government was granted to the Women’s to develop, and then distribute 2000 *Growing Together* kits and evaluate the outcomes. A team from Judith Lumley Centre (JLC) at La Trobe University in Melbourne, Australia, was engaged to undertake an evaluation of *Growing Together*, measuring various outcomes including women’s experience of motherhood, attachment, infant development, and parenting confidence as well as the views and experiences of health professionals.

This paper describes the study protocol for the kit evaluation.

## Methods/design

We will undertake a two-arm cluster randomised controlled trial (RCT) to explore the impact of the *Growing Together* kit on women’s experience of motherhood and a range of parenting-related outcomes, and to compare these with the outcomes and experiences of women who received the usual hospital information (standard care). We will also explore parents’ views of the kit.

### Randomisation and recruitment

The Women’s has four geographically-based maternity care teams, each of which has one antenatal clinic day per week (Monday - Thursday). To reduce the risk of contamination, (as would be the case if individual women were randomised), individual clinic booking days will be randomised, so that all women booking on a randomised day will receive the kit. Individual randomisation is not pragmatic given the high potential for clinician errors (clinicians would have to change the information they give to individual women throughout any booking clinic). Additionally, women may notice other women receiving different information packages.

Randomisation will be undertaken by an independent statistician at the Judith Lumley Centre. Using a blocked randomisation technique, the clinic days will be randomly allocated throughout the recruitment period to either intervention or standard care. The randomisation schedule will be kept confidential, with only the project coordinator, PhD student and senior research midwife having access in order to plan staffing for the research midwives.

As it is clusters (i.e. clinic days) being randomised and not individuals, randomisation will be done in advance, to ensure equal proportions of the four clinic days are randomly allocated to standard care and the intervention over the estimated 22 week recruitment period. The recruitment period was predicted based on the average number of booking visits per week at the Women’s and an estimation of women’s eligibility. For various reasons (e.g. geographical variations in the number of women meeting eligibility criteria), some clinic days may meet their quota of eligible women in fewer recruitment days than others. For this reason, recruitment will cease in each clinic (maternity care team) when their quota is reached.

### Eligibility criteria and processes

All eligible women booking for pregnancy care at the Women’s during the recruitment period will be included in the study. Inclusion criteria include primiparous women with adequate English-speaking ability (i.e. do not require an interpreter) and at ≤30 weeks gestation at first pregnancy booking appointment. Women will also be included if they had a previous stillbirth and no living children. The rationale for recruiting only primiparous women is that the information needs for first time parents are likely to be different than for parents who have already had a child.

**Fig. 1:**
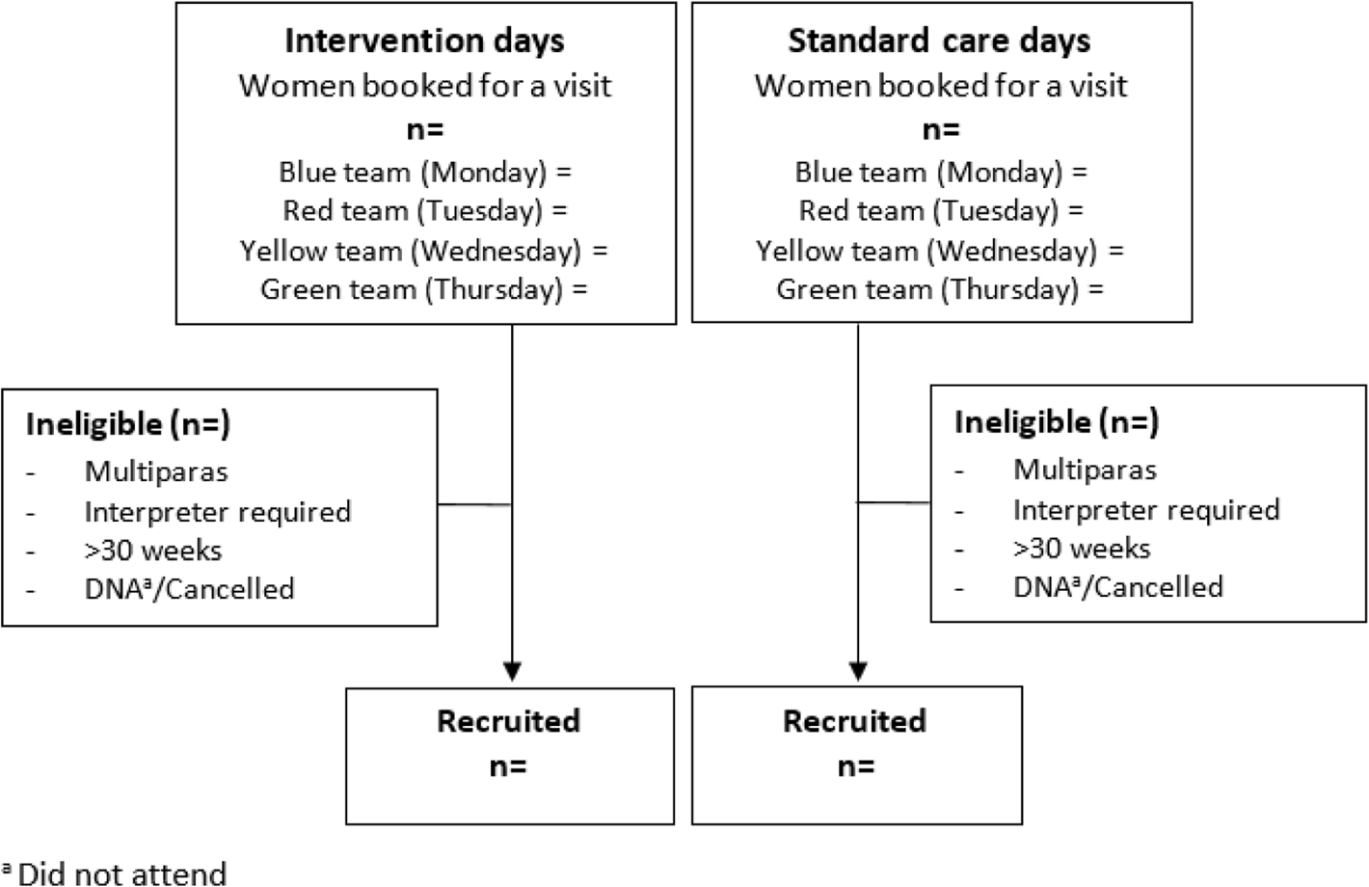
Recruitment of women to the trial arms. ^a^ Did not attend

To determine eligibility of women, prior to each randomised clinic day, the project coordinator or PhD student will screen all new booking histories to identify eligible women using the GP referral sheet, or available past history (Fig. 1). If identified as eligible, they are considered to be participants in the trial, in either the standard care or intervention arms as per the clinic randomisation schedule. Details of women who are identified as eligible (in either the intervention or standard care arms) will be entered into a password protected database with their estimated due date. These details will be used to facilitate the later checking of birth outcomes, mail-out of surveys and follow up phone calls at the specified data collection time points (described later). Births will be checked 2 weeks after the due date to ensure, as far as possible, that follow up contact occurs only with women who are discharged home with a live baby. The hospital’s electronic data systems will be used to facilitate this process.

### Description of standard care

Women in the standard care arm of the trial will receive the standard information resource at the Women’s, a booklet titled ‘*Having your Baby at the Women’s’*. This booklet includes information about care options at the Women’s and services and support provided during pregnancy and birth at the Women’s. It also includes information on how women should take care of themselves during pregnancy, information about different stages of pregnancy and relevant tests and screening as well as information for women to get ready for labour and birth. Care will be delivered in the usual way using standard resources, and these women will be followed up in order to provide a comparison group.

### Implementation of intervention

On the intervention clinic days, all eligible women will receive the *Growing Together* kit at their first booking visit. The kits will be attached to eligible women’s histories prior to clinic commencing, and will be provided by the midwives doing the booking visit. The research midwife will attend the clinic to ensure that the midwives responsible for the booking visits understand how to provide women with the kit. The research team will provide education about the RCT and the importance of providing the kit only on clinic days randomised to the intervention arm. No kits will be available or accessible outside of the intervention days.

Women who receive a kit will have a sticker added to the alert sheet in their medical record so that staff can continue to use and actively deliver the contents of the kit during subsequent pregnancy visits and beyond. It also means clinicians will know to ask about any concerns or questions in relation to the kit. Women having some pregnancy care with their GPs (referred to as *shared care*) will also have a sticker on their Victorian Maternity Record (VMR) documentation, which is the hand-held medical record that women carry with them to all their pregnancy visits. In addition, a letter explaining the *Growing Together* evaluation will be sent to GPs providing shared care with the Women’s Hospital.

During the RCT, the ‘App’ will be available only to women and their partners randomised to the intervention group. They will be mailed a brief reminder and instructions on how to download the App 1 week after the booking appointment. They will also be sent a letter and a sticker 1 week after birth, with instructions to add the sticker to the Child Health Record book and give the letter (explaining the *Growing Together* evaluation) to their Maternal and Child Health nurse (MCHN). The Women’s Hospital will also send out a letter explaining the *Growing Together* kit to the main local government area (LGAs) in the Women’s catchment area to be distributed to their MCHNs.

### Study outcomes

The study will evaluate a number of parenting related outcomes (Table 1), all to be compared by trial arm. The outcomes will be:

- Experience of motherhood.
- The experience of motherhood questionnaire (EMQ), a 20-item self-report measure [18] will be used as the primary outcome. The EMQ has been described as a coherent and valid measure of overall coping which is significantly related to and indicative of maternal depression, toddler temperament, social support and life events [18]. The EMQ scale scores range from 20 to 80, with lower scores indicating better maternal health and wellbeing.
- Parental health and emotional wellbeing.
- The Edinburgh Postnatal Depression Scale (EPDS) and Kessler 6 (K6) will be included in the survey to explore women’s and their partner’s emotional health and wellbeing. The EPDS is a 10-item self-report instrument [19]. Each item has four response options, scored from 0 to 3, with a total score between 0 and 30. The EPDS identifies women and partners likely to be experiencing postnatal depression, with scores ≥10 and > 12 indicating minor and major depression, respectively. K6 is a 6-item screening tool designed to identify emotional distress [20]. Items are rated on a five-point scales, ranging between 0 and 24. A score of ≥8 indicates non-specific distress and psychological symptoms, including depression and anxiety [21].
- Health-seeking behaviour for baby’s or mother’s own wellbeing.
- Developmental, emotional and physical health of babies (including infant feeding and infant temperament – sleeping, crying, settling) from birth to 12 months.
- To measure the baby’s temperament, four items relating to settling the baby (e.g. soothing) and the baby’s behaviour (e.g. crying) will be used. Each item scores from 1 to 3, with scores being averaged across four items [22]. Higher scores indicate more difficulties in settling and managing the baby.
- Parents’ knowledge and confidence.
- Parents’ confidence in conducting tasks related to caring for a baby will be measured using four items [23]. Scores to each item range from 1 to 10 which are summed and averaged to give a total score ranging from 1 to 10, with higher scores indicating greater confidence.
- Parental behaviour and parent-child interaction.
- Parent-child interactions will be assessed using four parenting dimensions, measuring ‘parenting self-efficacy’, ‘parental responsiveness/warmth’, ‘irritable parenting’ and ‘activities with child’ [22]. These measures were adapted for use in large scale surveys of Australian parents and demonstrated appropriate internal consistency, structural and predictive validity [23, 24], and sensitivity to the effects of parenting interventions [22].

**Table 1:** Data collection for the evaluation

	2 month survey	6 month interview	Partner survey	12 month survey
Views of the care and information received during pregnancy, birth and postnatal	X	X	X	
Views and experiences of the Growing Together kit (intervention)	X	X	X	X
Experience of motherhood – EMQ [18]		X		
Parental emotional wellbeing - EPDS [19] & K6 [20]	X	X	X	X
Health-seeking for baby’s or mother’s wellbeing		X		
Developmental, emotional and physical health of infant (e.g. infant feeding; infant sleeping; infant temperament [21])		X		X
Parents’ knowledge and confidence [22]		X	X	X
Parenting behaviour and parent-child interactions [21, 23]		X	X	X

‘Parenting self-efficacy’ will be measured using a single item global rating of the parent’s perception of themselves as being a ‘good’ parent, ranging from ‘1’ being a ‘not very good’ parent to ‘5’ being a ‘very good’ parent [22].

‘Parental responsiveness/warmth’ will be measured using six items from the Child Rearing Questionnaire [25] (scoring from 1 to 5), assessing parents’ expression of warmth, affection and enjoyment with the child [23]. ‘Irritable parenting’ will be measured using five items from Parental Perceptions and Behaviours Scale [26] (scoring from 1 to 5) about frequency of parental anger and irritability toward the child [23].

For these measures, item scores will be summed and then averaged making a total score ranging from 1 to 5 with higher scores indicating higher levels of responsiveness and irritability. Previous research reports internal consistency coefficients of .86 and .87 respectively when used with Australia mothers of infants and young children [21]. ‘Activities with child’ measures the extent to which parents engage in activities with their baby which are likely to support baby development. This will be assessed with four items, asking the frequency of activities with a baby in a typical week, rated on a 4-point scale from 1=‘not at all’ to 4 = ‘every day’ ((α = .71) [21].

- Parents’ views and experiences of the care and information received during pregnancy, birth and postnatal period.
- Parents’ views and experiences of the *Growing Together* kit and its usefulness (intervention arm only).
- Respondents’ background characteristics will also be collected in all surveys and interviews.

### Sample size

The experience of motherhood questionnaire (EMQ), a 20-item self-report measure, has been used to determine the sample size [18] with the goal of reducing the proportion of women scoring low on the EMQ. A score of 40 on the EMQ is a typical median score for a sample of first time Australian mothers. We therefore expect 50% of women in the standard care to score 40 or less. To detect a difference of 10% (50% compared to 60%) scoring 40 or less between new mothers who have received the kit and those who have not would require 408 per group (total of 816 women) with 95% confidence and 80% power. Allowing for loss to follow up we therefore aim to recruit 1000 mothers. We do not anticipate needing to allow for any potential effect of clustering given we will ensure relatively equal numbers of women from the four geographically-based teams (i.e. the main factor which may introduce differences) will be allocated to both trial arms. Similarly, the clinical guidelines, models of care, and appointment length are all uniform across the four teams, thus minimising the potential for different influences based on team allocation. Where appropriate, we will adjust for demographic and birth outcomes.

### Data collection

**Fig. 2:**
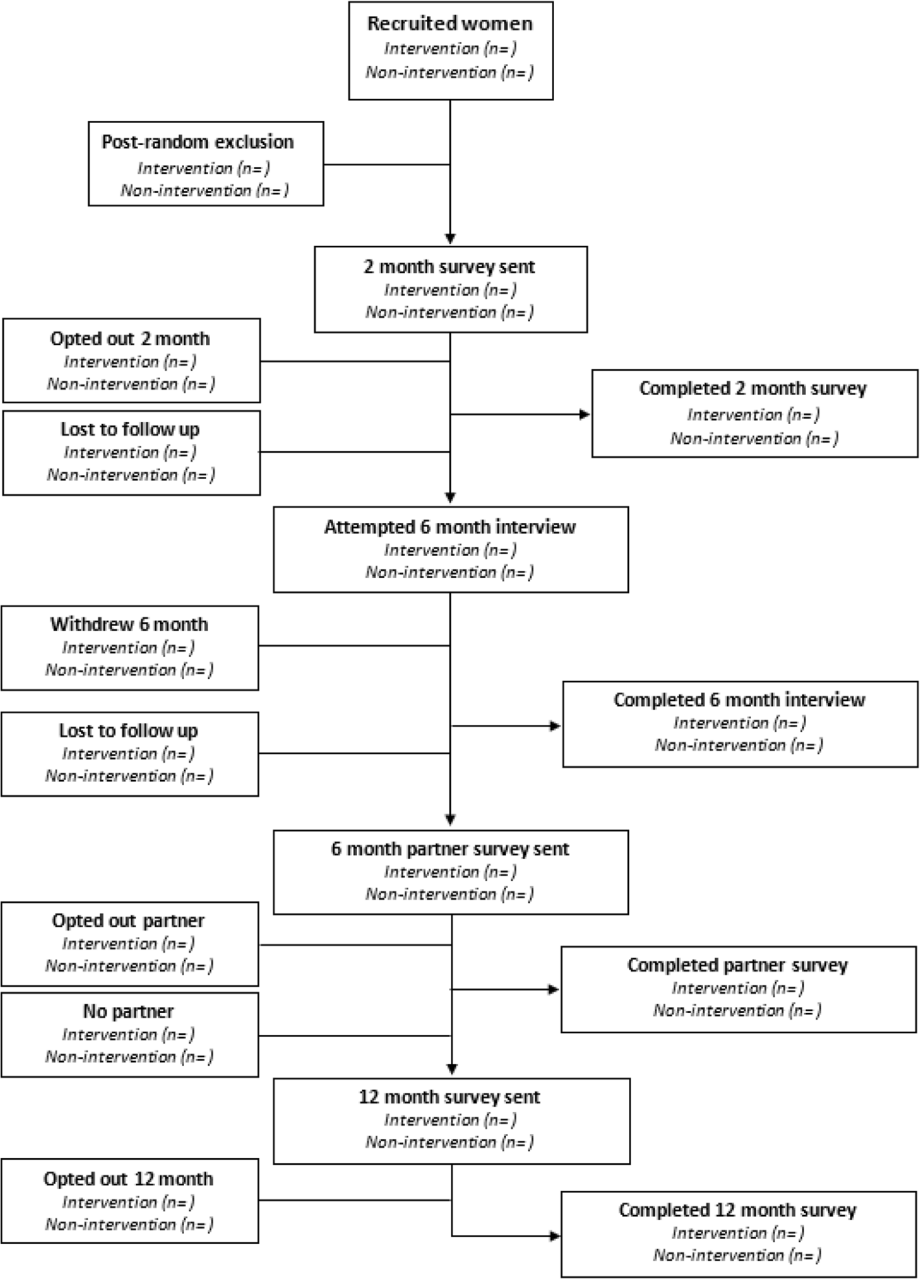
Data collection processes

The *Growing Together* kit covers the time period from conception until the baby reaches 12 months of age. Thus, we will evaluate the impact of the kit until this time. Outcome data will be collected from women and their partners at the following time points (Fig. 2):

- 2 months postpartum – postal survey with an option of online survey;
- 6 months postpartum – telephone interview;
- 6 months postpartum – partner postal survey with an option of online survey;
- 12 months postpartum – online survey.

#### 2 month survey

After the estimated date of birth of the baby, the hospital’s electronic data system will be checked for the birth outcome, and to ascertain that a live baby is taken home by the mother. Where a baby has died (either in utero or following birth, and prior to discharge from hospital), or where a baby is transferred for ongoing specialist neonatal care, women will be deemed a post randomisation exclusion, and receive no further contact as part of the evaluation. Women who do not continue their pregnancy care at the Women’s, or birth elsewhere will also be considered a post randomisation exclusion.

All remaining eligible women (in both the intervention and standard care arms) will be sent a postal survey when their baby is 2 months of age. A covering letter explaining the evaluation, an 'opt out' form, and two reply paid envelopes will be sent with the survey. The option to opt out will be explained and there will be a separate reply paid envelope in which women can return the form to a hospital midwife to remove their contact details from the survey mail out list. There will also be an option to complete the survey online, with a link included in the cover letter and reminder card. Completion and return of the survey will be considered as informed consent.

The 2 month survey will explore women’s views and experiences of the care and information they received during their pregnancy, birth and postnatal period, as well as their health and emotional wellbeing (Table 1). Women in the intervention arm will also be asked about their views and experiences of the *Growing Together* kit and its usefulness. The 2 month survey will take approximately 20 min to complete.

If women do not return the survey, they will be sent a reminder card 2 weeks later and another copy of the survey 4 weeks after the original survey is sent (if still no response). Women who opt out will not be sent any further reminders and will have no further follow up. Women will be marked as ‘non respondent’ (i.e. lost to this follow-up) if they do not return the survey by the time of data analysis. However, if they do not opt out from the study, they will still be included in the next data collection phase. To assess the mental health of women in the study, returned surveys will be checked for any concerning responses to the EPDS. Women who score high (≥13) or respond positive to the self-harm question will be contacted by an experienced midwife by telephone, as per a protocol to be developed for management of these women.

#### 6 month telephone interview

All eligible women (in both the intervention and standard care arms) will be telephoned by a research midwife when their baby is 6 months of age, to conduct an interview. Women who had previously been excluded or who opted out will not be contacted.

As per Table 1, the 6 month telephone interview will explore: women’s experience of motherhood and caring for their baby; maternal-infant attachment; developmental, emotional and physical health of the baby (including infant feeding and infant temperament – sleeping, crying, settling) and any visits to hospital from birth to 6 months; as well as women’s own wellbeing and health-seeking behaviour since birth. Women in the intervention arm will also be asked about their use of the *Growing Together* kit since birth, and its usefulness. The 6 month telephone interview will take approximately 30 min to complete.

Attempts to contact women will be made as per a structured telephone interview protocol. Where a telephone has been disconnected or a number has changed, the hospital electronic data system will be checked for an updated number. If a woman remains uncontactable by the time her baby is 9 months old (i.e. after 3 months of attempted contact) she will be deemed ‘lost to follow up’ at 6 months. An SMS, asking women to contact us regarding a suitable time for a follow up call, will also be sent 2 to 3 weeks prior to the 9 month cut off as an alternative mode of contact.

#### 6 month partner survey

A postal survey will be sent (via women) to the partner of all eligible women (in both the intervention and standard care arms) when their baby is 6 months of age. If the survey is not returned, a reminder card will be sent 3 weeks later, and another copy of the survey 5 weeks after the original survey has been sent. The partner survey can also be completed online via a link which will be included in the initial covering letter, reminder card and final reminder letter.

The cover letter will include an option for partners to opt out if they would prefer not to be involved in the study. It will also include a section for women who either never had, or no longer have, a partner to stipulate that they have ‘no partner’. If women have ‘no partner’ or partners opt out, they will be removed from the list of partners for further follow up.

The survey will explore the woman’s partner’s views and experiences of information and care they or their partner received during their partner’s pregnancy and in the period following the birth of their baby (Table 1). They will also be asked about their experience of parenting, caring for their baby, parenting behaviour and paternal-infant interaction as well as about their emotional health and wellbeing. The partner survey will take approximately 10 min to complete.

Similar to the 2 month survey, returned partner surveys will be checked for responses to the EPDS questions and the protocol will be followed if a partner scores high on the EPDS (≥13) or responds positive to the self-harm question.

#### 12 month survey

All eligible women will be sent an SMS with an explanation of the survey and a link directly to the 12 month online survey, when their baby is 12 months of age. The online survey will be able to be completed on the woman’s mobile device. Women will have the opportunity to opt out, and there will be a reminder system in place, sending SMS reminders at 2 weeks and 4 weeks from the initial SMS.

The 12 month follow up will be a short online survey and will take approximately 10 min to complete. It will explore women’s parenting outcomes, their baby feeding and temperament as well as their emotional wellbeing and ongoing use of the kit (in the intervention arm) (Table 1).

### Data management and analysis

Survey data will be entered into the REDCap data management system [27]. All data will be then transferred to Stata version 14 [28] for data cleaning and analysis.

Data accuracy will be checked through several steps, including verification of the missing data, the range and format of values and checking consistencies and frequency distributions. Any missing or inconsistent values will be checked with the hard copy surveys.

In relation to the trial outcomes, trial arms will be compared using intention to treat analysis. Randomising the various clinic days is likely to result in groups that are evenly distributed - however analyses of study outcomes will be adjusted for baseline differences between the groups as needed. The first stage of the analysis will describe the demographic characteristics in the two trial arms using numbers and percentages, and these data will enable us to understand what factors we need to take into account in the analysis of outcomes. Study outcomes will be compared using χ^2^ and relative risks, with an additional multivariate analysis where there are differences in demographic characteristics of the women in the two trial arms. Comparison of means will be undertaken for continuous variables using t-tests where data are normally distributed or medians compared otherwise using Mann-Whitney U tests.

If the proportions of missing data are above 5%, several steps will be considered to handle missing data. The missing values will be first explored by trial arm and based on the characteristics of the missing data to identify if missing data are missing completely at random (MCAR), missing at random (MAR) or missing not at random (MNAR). Multiple imputation will be conducted considering reasons for and amount of missing data, availability of predictors of missingness and variables that are correlated with the incomplete variable. If the proportions of missing data are very large (more than 40%), only the results of the complete case analysis will be reported and the interpretative limitations of the trial results will be mentioned [29].

### Process evaluation measures

A number of strategies will be considered for process evaluation and to evaluate intervention implementation and uptake.

On the intervention recruitment days research midwives will be present to monitor the number of kits given. Kits will be attached to the history of eligible women; there will be only a limited number of kits distributed each day according to the pre-determined number of eligible women.

#### Measures of intervention exposure

Women in the intervention arm will be asked if they were given the *Growing Together* kit at their pregnancy visit. They will also be asked about their use of the kit during pregnancy, birth and after birth; and if midwives and/or doctors used the kit during their antenatal appointments.

In addition, the views and experience of midwives and medical staff providing maternity care at the Women’s will be explored. They will be asked if they knew about the kit and whether they used the kit at antenatal appointments during the trial period.

### Ethical considerations

Given clinic days will be randomised, individual consent will not be sought; rather potential respondents will be informed as described below.

When the first survey (at 2 months postpartum) is mailed out, the cover letter will explain that the Women’s is interested in evaluating the information they provide to prospective and new parents. It will explain that as part of the evaluation, women will be sent a survey, will be contacted at 6 months post-birth for a short telephone interview and then will be sent a final survey 12 months after the birth. The letter will also explain that they will receive a postal survey for partners to complete at around 6 months. If women prefer not to be involved they may complete an ‘opt out’ form. We will consider return of the surveys as ‘consent’, and if women do not opt out they will be included in the ongoing follow up.

The cover letter will explain that JLC has been engaged to undertake the work, and that the surveys have been sent by the Women’s Hospital (to reassure women regarding any privacy concerns). The cover letter will include contact details for research team if parents have any questions or would like to discuss the study. For women who choose to opt out of receiving further follow up, they will be able to send the opt out form back to a Women’s staff member via a pre-addressed reply paid envelope, and their contact details will be removed from the survey mail out list. For women who wish to participate, they will be able to return their completed survey in a pre-addressed, reply paid envelope to the research team.

We prefer this ‘opt out’ method as opposed to an ‘opt in’ given our knowledge of the response rates to postal surveys among similar women – often 30 to 40% in surveys we have conducted at the Women’s [30] and elsewhere in Victoria [31]. If we required women to ‘opt in’, the chance of having this percentage of respondents would probably be even lower, and thus diluting the generalisability of the findings. Participants will not be offered any type of incentives for completion of the surveys.

Prior to sending out the surveys after the birth, we will check that only women who are discharged with a live baby are on the list. Where women have a known antenatal or neonatal loss they will be excluded, and women’s contact details will be removed from the survey mail out list.

Research Ethics approval has been obtained from the Women’s Hospital (RWH HEC no 14–44). The project was subsequently approved by La Trobe University UHEC, as an ‘externally approved project’ based on approval of the Women’s (UHEC acceptance of RWH HREC approved project – 14/44).

### Dissemination

Three reports will be submitted to the Women’s, to follow the three stages of data collection. The first report will include data from the 2 month survey, and the second will include data from the 6 month telephone interview and partner survey. The final report will be submitted after completion of the 12 month survey. Study findings will be presented in national and international conferences, in peer reviewed publications and in a higher degree thesis.

### Timelines

**Fig. 3:**
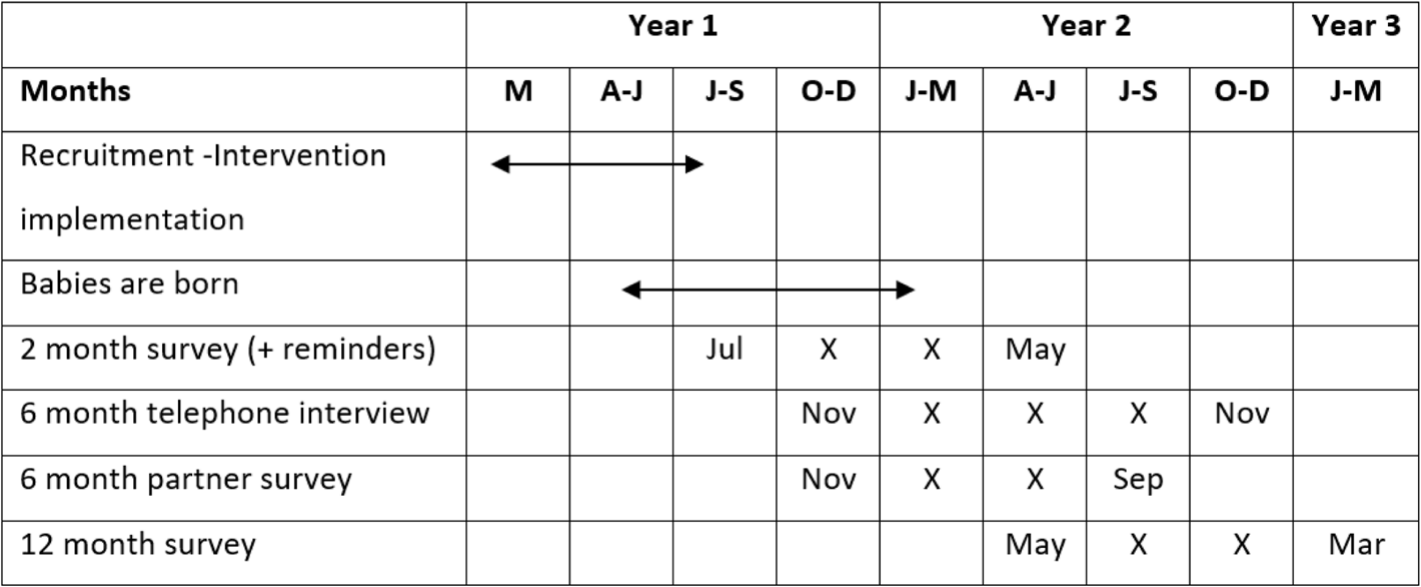
Timeline estimate for the trial

The trial is expected to take 3 years from implementation of the intervention to completing data collection (Fig. 3).

## Discussion

Prospective and new parents would like trusted and quality information to prepare them for parenting. This evaluation will provide valuable information in terms of the views and experiences of women and their partners regarding the kit and its usefulness. It will also determine the impact of the kit on a range of outcomes including women’s experience of motherhood, infant development and parental confidence.

## Data Availability

Not applicable.

## Abbreviations

EMQ: Experience of Motherhood Questionnaire
EPDS: Edinburgh Postnatal Depression Scale
GP: General Practitioner
JLC: Judith Lumley Centre
K6: Kessler 6
LGA: Local Government Area
MCHN: Maternal and Child Health Nurse
RCT: randomised controlled trial
SMS: Short Message Service
the Women’s: the Royal Women’s Hospital
VMR: Victorian Maternity Record
